# Deciphering *Escherichia coli* ESBL/pAmpC Plasmids Through High-Throughput Third-Generation Sequencing and Hybrid Assembly

**DOI:** 10.3390/pathogens14101039

**Published:** 2025-10-13

**Authors:** Andrea Laconi, Enea Ovedani, Roberta Tolosi, Ilias Apostolakos, Alessandra Piccirillo

**Affiliations:** 1 Department of Comparative Biomedicine and Food Science, University of Padua, 35020 Legnaro, Italy; enea.ovedani@studenti.unipd.it (E.O.); roberta.tolosi@unipd.it (R.T.); alessandra.piccirillo@unipd.it (A.P.); 2 Veterinary Research Institute, Hellenic Agricultural Organization “DIMITRA”, 57001 Thessaloniki, Greece; iaposto@hotmail.com

**Keywords:** hybrid assembly, MinION, Illumina, plasmid, AMR, ESBL, pAmpC, insertion sequence

## Abstract

Extended-spectrum β-lactamases (ESBLs) and plasmid-mediated AmpC (pAmpC) β-lactamases represent a threat for public health. Their dissemination is often mediated by mobile genetic elements (MGEs), but plasmid identification and characterization could be hindered by sequencing limitations. Hybrid assembly may overcome these barriers. Eight ESBL/pAmpC-producing *E. coli* isolates from broilers were sequenced using Illumina (short-read) and Oxford Nanopore MinION (long-read). Assemblies were generated individually and using a hybrid approach. Plasmids were typed, annotated, and screened for antimicrobial resistance genes (ARGs), MGEs, and virulence factors. Short-read assemblies were highly fragmented, while long reads improved contiguity but showed typing errors. Hybrid assemblies produced the most accurate and complete plasmids, including more circularized plasmids. Long and hybrid assemblies detected IS26 associated with ESBL genes and additional virulence genes not identified by short reads. ARG profiles were consistent across methods, but structural resolution and contextualization of resistance loci were superior in hybrid assembly. Hybrid assembly integrates the strengths of short- and long-read sequencing, enabling accurate plasmid reconstruction and improved detection of resistance-associated MGEs. This approach may enhance genomic surveillance of ESBL/pAmpC plasmids and support strategies to mitigate antimicrobial resistance.

## 1. Introduction

Bacterial resistance to antimicrobial agents is a major threat to public health [1]. Cephalosporins, a class of β-lactam antibiotics, are widely used in both human and veterinary medicine. Third-generation cephalosporins (3GCs) have particular therapeutic value in human medicine and represent one of the limited treatment options for infections caused by multidrug-resistant Enterobacteriaceae [2]. Resistance to these agents is frequently mediated by extended-spectrum β-lactamases (ESBLs) and AmpC β-lactamases (AmpCs). The genes encoding these enzymes are often located on plasmids and associated with mobile genetic elements (MGEs), such as transposons (Tns) and insertion sequences (ISs) [3,4]. Consequently, horizontal gene transfer (HGT) via mobilization or conjugation plays a major role in the dissemination of β-lactam resistance across different strains and genera of the Enterobacteriaceae family [5]. Among them, *Escherichia coli* is a key player in the spread of antimicrobial resistance (AMR), as it readily acquires and transfers AMR genes (ARGs) to other bacterial species [6]. Thus, genetic characterization of plasmids and their associated MGEs in *E. coli* is essential for understanding the molecular mechanisms driving AMR dissemination [6].

Whole-genome sequencing (WGS) provides a powerful tool for analyzing bacterial genomes and investigating ARGs [7]. Illumina short-read sequencing remains the most widely used technology in microbial genomics due to its high accuracy and throughput [8]. However, reconstructing plasmids from short reads alone is challenging, as repetitive regions often exceed the read length and typical paired-end insert sizes (~300–500 bp), which prevents complete plasmid assembly and hinders accurate contextualization of ARGs [9,10]. In contrast, long-read sequencing platforms such as Oxford Nanopore Technologies (ONT) MinION generate reads of 8–10 kb or longer, which can span repetitive regions and improve plasmid reconstruction. Despite their higher error rates (5–15%), ongoing technological advances are steadily reducing these limitations. Hybrid assembly, which combines the structural resolving power of long reads with the base-level accuracy of short reads, has emerged as a promising approach for generating accurate and contiguous plasmid assemblies [10].

This study aimed to assess whether hybrid assembly enhances the characterization of ESBL/pAmpC-carrying plasmids in *E. coli* isolated from the broiler production pyramid compared to short- or long-read sequencing alone. In particular, we evaluated the ability of each approach to resolve plasmid structures, identify co-localized ARGs, characterize MGEs, and detect virulence genes.

## 2. Materials and Methods

### 2.1. Bacterial Isolates

The eight ESBL/pAmpC-producing *E. coli* strains included in this study (Table 1) were isolated from three production chains (A, B, and C) of an integrated broiler company in Northern Italy [11]. Screening for ESBL/pAmpC was performed on Eosin Methylene Blue agar (Microbiol, Cagliari, Italy) supplemented with 1 mg/L cefotaxime (CTX-EMB) and incubated at 37 ± 0.5 °C for 20 ± 2 h. ESBL/pAmpC production was confirmed by the double-disk synergy test according to CLSI guidelines [12]. Isolates characterization (i.e., phylogroups and sequence typing) and detection of ESBL/pAmpC resistance genes were performed by multiplex PCR [11]. A subset of isolates, selected on the basis of production chain, production stage, ESBL/pAmpC gene, and phylogroup combinations, was sequenced using Illumina short-read [13].

### 2.2. Library Preparation and Whole-Genome Sequencing

In a previous study [13], bacterial DNA was extracted using the Invisorb Spin Tissue Mini Kit (Invitek, Berlin, Germany), libraries were prepared using the Nextera XT library preparation kit (Illumina, San Diego, CA, USA), and sequencing was performed on an Illumina HiSeqX platform with 2 × 150 bp paired-end reads (Macrogen, Seoul, Republic of Korea). In the present study, bacterial DNA was extracted with the QIAprep Spin Miniprep Kit (Qiagen, Hamburg, Germany) and long-read sequencing was performed on an ONT MinION platform using the Rapid sequencing gDNA barcoding kit (SQK-RBK110.96) (ONT, Oxford, UK) and R9.4.1 flow cells (ONT, Oxford, UK).

### 2.3. Raw Reads Quality Control

**Figure 1:**
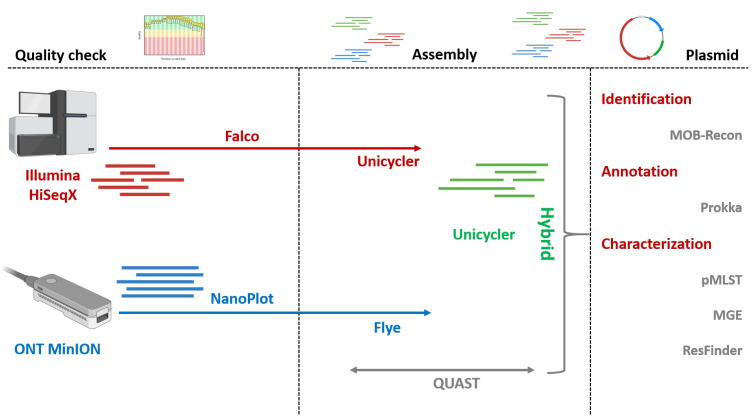
Schematic representation of the bioinformatics workflow applied for sequencing data processing, genome assembly, plasmid reconstruction, and downstream analyses.

Illumina read quality was assessed using Falco (v1.2.4) [14]. Adapters and low-quality reads (Phred < 25) were removed using Trimmomatic (v0.39) [15] with default parameters. MinION reads were evaluated with NanoPlot (v1.44.1) [16] and filtered (Q > 8) with Nanofilt (v2.3.0) [17]. A graphical representation of the entire workflow is depicted in Figure 1.

### 2.4. Bacterial and Plasmid Whole-Genome Assembly

Short-reads assemblies were generated using Unicycler (v0.5.1) [18], meanwhile MinION long-read sequences were assembled using Flye (v2.9.6) [19]. Hybrid assemblies integrating both short- and long-read data were obtained using Unicycler (v0.5.1). General assembly statistics and quality metrics of the assembled genomes were calculated using QUAST (v5.3.0) [20].

### 2.5. Identification, Annotation, and Characterization of ESBL/pAmpC-Carrying Plasmids

MOB-Recon (v3.1.9) [21] was used to reconstruct and type individual plasmid sequences from short-, long-read, and hybrid assemblies. Plasmids harboring ESBL/pAmpC genes were characterized using pMLST 2.0 (v0.1.0) with default parameters [22] and annotated with Prokka (v1.14.6) [23]. ISs and Tns were identified using MGE (v1.14.6) with default parameters [24].

### 2.6. Resistance and Virulence Genes Detection

ARGs located on ESBL/pAmpC-carrying plasmids and other plasmids were identified using ResFinder (v4.7.2) with 90% identity threshold and 60% coverage [25]. Virulence genes were detected using MGE (v1.14.6) [24].

### 2.7. Statistical Analysis

Differences in assembly performance (e.g., number of contigs, total length, N50, number of genetic features extracted) across short-read, long-read, and hybrid assemblies were assessed using the non-parametric Kruskal–Wallis test followed by Dunn’s post hoc test using GraphPad Prism (v10.5.0). Significant differences were set at a *p*-values < 0.05.

## 3. Results

### 3.1. Basic Statistics of Short and Long Reads

Statistics for both Illumina and MinION reads after trimming and quality filtering are reported in Table 2.

**Figure 2:**
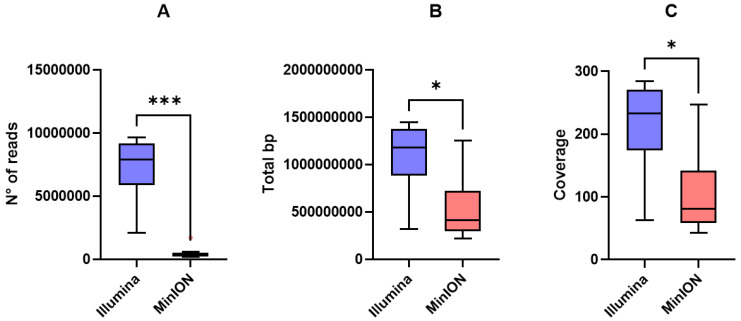
Tukey box plots of (**A**) number of reads, (**B**) total bp, and (**C**) genome coverage yielded by Illumina short-reads (blue) and MinION long-reads (red). Differences were assessed using the non-parametric Kruskal–Wallis test followed by Dunn’s post hoc test. *p* < 0.05 is shown as * and *p* < 0.001 as ***.

EC-33 and EC-91 showed higher long-read coverage compared to the other genomes. Meanwhile, short-read coverage was comparable among strains. Illumina reads yielded significantly higher number of reads (*p* = 0.0002), total base pair (bp) (*p* = 0.01), and coverage (*p* = 0.01) compared to long-reads (Figure 2A–C).

### 3.2. Performances of Short, Long, and Hybrid Assemblies

**Figure 3:**
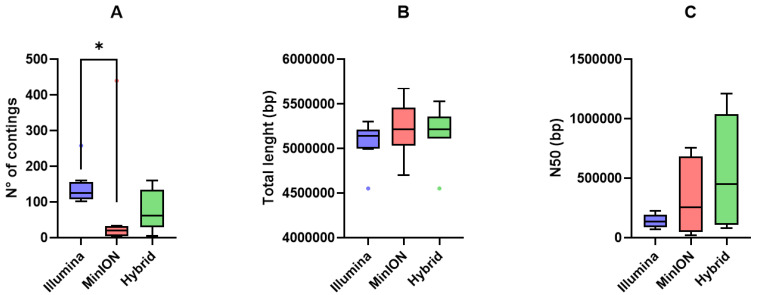
Tukey box plots of (**A**) number of contigs, (**B**) total length (bp), and (**C**) N50 (bp) yielded by Illumina short-reads (blue), MinION long-reads (red), and hybrid assemblies (green). Differences were assessed using the non-parametric Kruskal–Wallis test followed by Dunn’s post hoc test. *p* < 0.05 is shown as *.

An overview of the comparison of the genome assembly performances of short-, long-read, and hybrid assemblies is reported in Figure 3.

Illumina short-reads produced more fragmented assemblies (higher number of contigs) compared to MinION long-reads and hybrid assemblies (*p* < 0.05; Figure 3A). Total assembly length (bp) was comparable among the three approaches (Figure 3B), whereas N50 values were higher for long-read and hybrid assemblies than for short-read assemblies (Figure 3C).

### 3.3. Identification, Annotation and Characterization of ESBL/pAmpC-Carrying Plasmids in Short, Long, and Hybrid Assemblies

**Figure 4:**
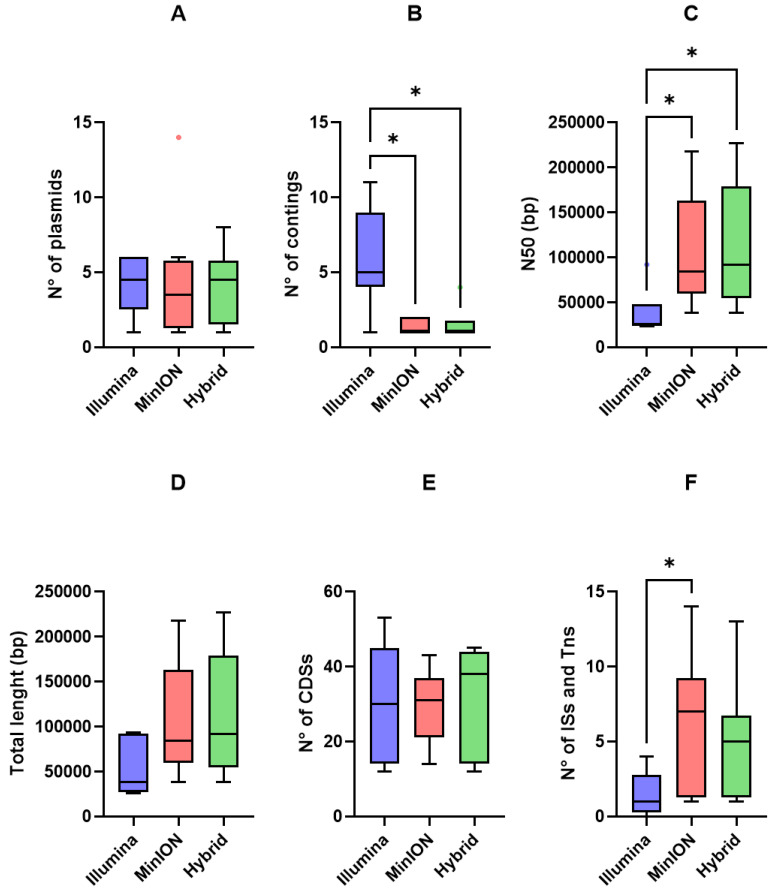
Tukey box plots of (**A**) number of plasmids, (**B**) number of contigs, (**C**) N50 (bp), (**D**) total length (bp), (**E**) number CDSs, and (**F**) number of IS and Tn sequences yielded by Illumina short-reads (blue), MinION long-reads (red), and hybrid assemblies (green). Differences were assessed using the non-parametric Kruskal–Wallis test followed by Dunn’s post hoc test. *p* < 0.05 is shown as *.

MOB-Recon detected a similar number of plasmids across assemblies (Figure 4A) and correctly identified the plasmid carrying the ESBL/pAmpC genes for each strain across the three assembly approaches.

Some differences among the assemblies were observed. Illumina assemblies produced a higher number of contigs compared to MinION (*p* = 0.033) and hybrid (*p* = 0.016) assemblies (Figure 4B), indicating that Illumina reads generated more fragmented plasmid sequences. Accordingly, N50 values were significantly higher for MinION (*p* = 0.044) and hybrid (*p* = 0.048) assemblies than for Illumina assemblies (Figure 4C). Lower contig numbers and higher N50 values corresponded to larger average plasmid sizes, although the differences were not statistically significant (*p* > 0.05; Figure 4D). While short- and long-read assemblies produced only one and two ESBL/pAmpC circular plasmids, respectively, four ESBL/pAmpC plasmids were fully circularized in hybrid assemblies (Table 3).

**Figure 5:**
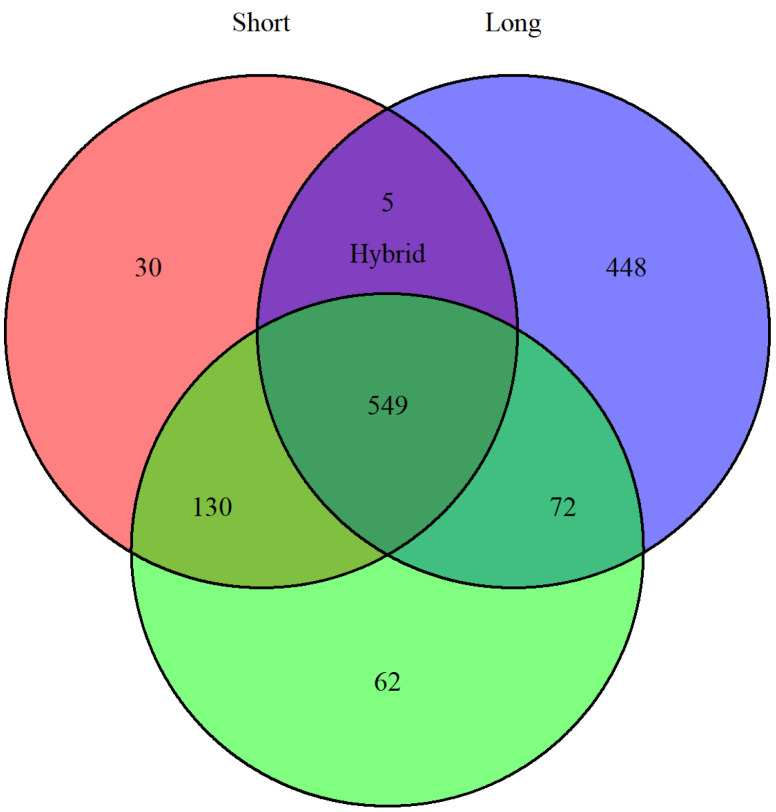
Venn diagram prepared with VennDiagram in R (v4.5.1) plotting the differences and overlaps of annotated CDSs (including putative and hypothetical proteins) for short (red), long (blue), and hybrid (green) assemblies.

Seven different Inc replicons were identified across the eight plasmids (Table 3). MinION and hybrid assemblies detected all replicons, whereas Illumina reads failed to identify the IncFII replicon in the plasmid from strain EC-94. pMLST analysis was performed for replicons IncA, IncI1, and IncHI2. MinION assemblies performed the least effectively, failing to assign a plasmid sequence type (pST) for EC-94 and producing mismatches or gaps for the other plasmids. pMLST profiles matched between Illumina and hybrid assemblies, except for the plasmid in EC-40; Illumina classified it as pST-26 (clonal complex CC2), whereas hybrid assemblies identified it as a new pST assigned to CC-26. In both cases, pMLST alleles were perfectly matched to known references. Across all assemblies, 1296 coding DNA sequences (CDSs), including putative and hypothetical proteins, were identified among the eight plasmids. Annotated CDSs from Illumina and hybrid assemblies showed strong overlap (Figure 5), while MinION assemblies produced divergent results.

When putative and hypothetical proteins were excluded, short-, long-, and hybrid assemblies yielded a comparable number of CDSs (Figure 4E). Long assemblies showed higher performances in identifying IS and Tn sequences compared to Illumina reads (mean = 6.13 vs. mean = 1.5, and *p* = 0.042, Figure 4F). Furthermore, long-read and hybrid assemblies overperformed short-reads in characterizing the relationship between MGEs and ESBL/pAmpC genes. Indeed, while Illumina identified only three ISs (i.e., ISEc9 and IS26) and one Tn (Tn2) associated with ESBL/pAmpC genes, MinON and hybrid identified six ISs (i.e., ISEc9, IS26, and IS102), one Tn (Tn2), and one composite Tn (cn_5325_IS26) (Table 4).

**Figure 6:**
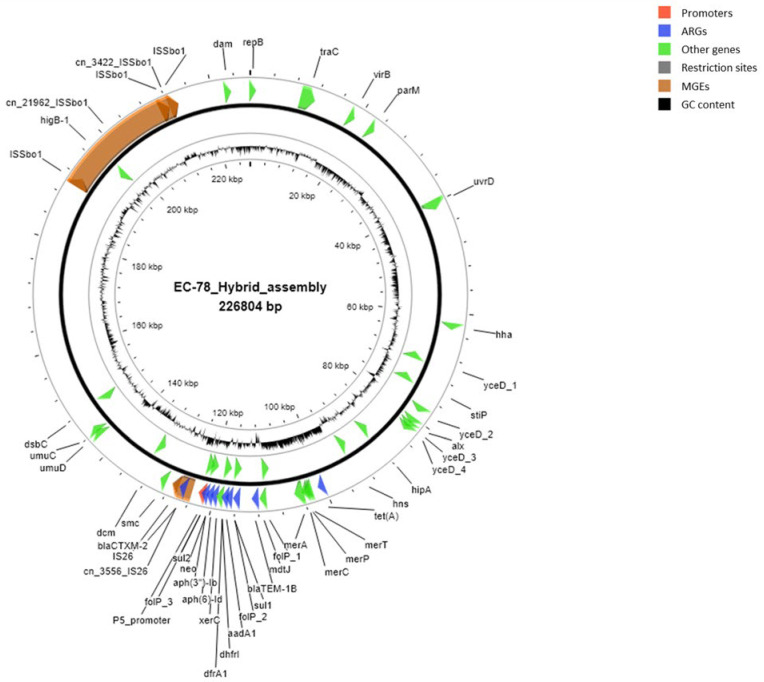
Map of the ESBL-carrying plasmid harbored by strain EC-78, based on sequences and annotations obtained using the hybrid assembly approach. Mobile genetic elements (MGEs) are shown in orange, antimicrobial resistance genes (ARGs) in blue, other genes in green, and promoters in red. GC content and plasmid size are also indicated. Putative and hypothetical proteins are not reported.

A map of the ESBL-carrying plasmid harbored by strain EC-78, based on sequences and annotations obtained using the hybrid assembly approach, is shown in Figure 6.

### 3.4. Identification of Acquired Antimicrobial Resistance Genes and Virulence Factors in Short, Long and Hybrid Assemblies

Aside from genes against 3GCs, 25 different ARGs conferring resistance to eight antimicrobial classes (i.e., aminoglycosides, β-lactams, (fluoro)quinolones, macrolides, phenicol, sulphonamides, tetracyclines, and trimethoprim) were identified by the three assemblies across the eight ESBL/pAmpC carrying plasmids. According to all three assemblies, five out of eight plasmids showed a multi-resistant profile, carrying genes conferring resistance to three or more antimicrobial classes. Detail of ARGs identified by each assembly for each plasmid are reported in Table 4. Six known virulence genes (i.e., cib, terC, traJ, traT, astA, and anr) distributed among four ESBL/pAmpC plasmids were identified by long and hybrid assemblies, while short read detected only four.

### 3.5. Characterization of Other Plasmids in Short, Long and Hybrid Assemblies

Plasmids other than those carrying the ESBL/pAmpC genes were identified in all the strains but one (i.e., EC-91). The average plasmids size was comparable among the three assemblies (Figure S1A); however, hybrid assemblies yielded a higher number of circular plasmids compared to short- (*p* = 0.184) and long- (*p* = 0.129) read assemblies (Figure S1B). Nine different plasmid types (i.e., Col(MG828), ColpVC, ColRNAI, IncFIA, IncFIB, InCFIC, InchHI1B, IncHI2A, and IncI-gamma/K1) were identified across the short, long, and hybrid assemblies, with the latest showing the best typing performances (65.38%, 95% Confidence Interval (CI) 45.79–84.98% vs. 44.12%, 95% CI 26.53–61.70% and 55.56%, 95% CI 35.52–75.59%, respectively), even though the differences among assemblies were not significant (Figure S1C). In total 15 different ARGs belonging to seven different antimicrobial classes (i.e., aminoglycosides, β-lactams, macrolides, phenicol, sulphonamides, tetracyclines, and trimethoprim) were identified among the non-ESBL/pAmpC plasmids across the three assembly methods. Plasmids harbored by strains EC-56 and EC-94 showed multi-resistance profile. While long assembly identified a lower number of ARGs compared to the other two methods, no significant differences were observed (Figure S1D). Long and hybrid assemblies identified a higher number of ISs in non-ESBL/pAmpC plasmids (Figure S1E) compared to Illumina reads; indeed, while short-read assemblies detected 0.9 ISs per plasmid, this ratio increased to 1.48 and 1.85 for long and hybrid assemblies, respectively. Similarly, long and hybrid assemblies identified more virulence genes (*n* = 51 and *n* = 47, respectively) compared to short-read assembly (*n* = 40) (Figure S1F).

## 4. Discussion

The present study focused on the characterization of ESBL/pAmpC-carrying plasmids in *E. coli* strains isolated from the broiler production pyramid, using short-, long-, and hybrid-read assembly approaches. Our results, consistent with previous observations, demonstrate that the hybrid assembly strategy outperformed the exclusive use of either Illumina (short-read) or MinION (long-read) data in resolving plasmid structures and enabling detailed characterization [8,10,26]. Illumina assemblies produced a higher number of contigs and lower N50 values than MinION and hybrid assemblies, resulting in more fragmented and smaller plasmids. Consequently, short-reads yielded fewer circularized plasmids, both ESBL/pAmpC-carrying and non-ESBL/pAmpC, compared with the other two approaches, particularly the hybrid assembly approach. These findings reflect a known limitation of Illumina sequencing; short read lengths cannot span repeated elements commonly found in plasmids (e.g., ISs and Tns), making plasmid reconstruction more difficult and less accurate [27]. Long-read assemblies overcame this limitation but highlighted the lower sensitivity and higher error rate of nanopore sequencing. Specifically, long-read assemblies performed poorly in plasmid typing, producing mismatches and gaps against reference sequences and failing to type most plasmids, suggesting that nanopore data alone may not be optimal for plasmid typing or single-variant analysis [28]. However, in this study, long-reads assembly correctly classified two closely related genes of the *bla_CTX-M_* group (i.e., *bla_CTX-M-1_* and *bla_CTX-M-15_*), which shared a nucleotide sequence similarity of about 98.7% [29]. In hybrid assemblies, Illumina data corrected MinION errors, while preserving the comprehensive detection of ISs and Tns. As a result, hybrid assemblies achieved typing accuracy comparable to Illumina while resolving plasmid structures more effectively and producing the highest number of circularized plasmids. This enhanced resolution enabled the characterization of gene clusters carrying resistance determinants and transfer systems, highlighting potential recombination mechanisms mediated by insertion sequences and other transposable elements [30]). Long-reads and hybrid assembly enabled to identify the association between three different ESBL genes (i.e., *bla_SHV-12_*, *bla_CTX-M-1_*, and *bla_CTX-M-2_*) and IS26, which would have been missed if using Illumina sequencing alone. Because IS26 plays a key role in the mobility of ESBL genes between plasmids and from plasmids to the chromosome [31], the absence of this information can hinder our understanding of ESBL gene transmission across humans, animals, and the environment. Indeed, a recent study combining the genetic analysis of more than 2500 plasmid sequences with in vitro inter-plasmid antibiotic resistance gene transfer experiments reported that IS26 is likely to accelerate ARG dissemination among different bacterial species [32]. Furthermore, IS26-mediated transposition activity of *bla_KPC-2_* seems to play a key role in the emergence of carbapenem resistance in *Klebsiella pneumoniae* [33]. Therefore, the identification and characterization if ISs, in particular IS26, are essential for implementing effective control measures and limiting the dissemination of critically important ARGs. Although dual sequencing increases costs, from an epidemiological and surveillance perspective, this added resolution justifies the use of hybrid assemblies.

The three approaches identified similar, if not identical, ARG profiles across the eight ESBL/pAmpC plasmids, indicating that hybrid assembly does not markedly improve resistance gene detection, in contrast to some previous observations [34].

VF gene prediction was comparable between long-read and hybrid assemblies, whereas Illumina identified fewer VF genes in both ESBL/pAmpC and non-ESBL/pAmpC plasmids. Previous studies have reported conflicting results when comparing short- and long-read sequencing for virulence gene detection [27,35]. Such discrepancies may be attributable to differences in library preparation, assembly, or bioinformatic tools and should be considered when comparing results across studies.

In conclusion, this study shows that hybrid assembly is a powerful approach in bacterial genomics. It provides a high-resolution view of plasmid architecture, improves the detection of resistance genes and their associated MGEs, and yields additional insights beyond those obtained from short- or long-read assemblies alone. Routine implementation of hybrid assemblies could generate essential knowledge to support targeted surveillance and intervention strategies against antimicrobial resistance.

## 5. Conclusions

This study highlights the advantages of hybrid assembly in resolving the structure and genetic context of ESBL/pAmpC-carrying plasmids from *E. coli* in the broiler production chain. By integrating short- and long-read sequencing, hybrid assemblies overcome the limitations of individual platforms, enabling more accurate plasmid reconstruction and the detection of key mobile genetic elements, such as IS26, involved in resistance gene dissemination. Although resistance gene profiles were broadly similar across approaches, the enhanced structural resolution provided by hybrid assemblies offers valuable insights into plasmid dynamics. Routine application of this strategy can strengthen antimicrobial resistance surveillance and guide more effective interventions within a One Health framework.

## Figures and Tables

**Table 1:** Phenotypic and genotypic features of the eight ESBL/pAmpC-producing *E. coli* included in the study.

Strain	Sample-Type	Production Stage	Phylogroup	Sequence Type (ST)	Serotype	ESBL/pAmpC Genes
EC-7	cloacal swab	breeders	C	88	O8:H4	*bla_CMY-2_*
EC-33	cloacal swab	breeders	A	695	H38	*bla_TEM-52B_*
EC-40	cloacal swab	broiler-chicks	B1	155	O120:H51	*bla_SHV-12_*
EC-56	cloacal swab	broilers	D	38	H15	*bla_CTX-M-1_*
EC-78	cloacal swab	broilers	A	4980	O88:H7	*bla_CTX-M-2_*
EC-91	carcass	carcass	D	69	O15:H18	*bla_CTX-M-15_*
EC-94	carcass	carcass	A	4937	O126:H38	*bla_CTX-M-1_*
EC-115	carcass	carcass	A	3107	O98:H12	*bla_CTX-M-1_*

**Table 2:** An overview of basic sequence information statistics and quality. Genome coverage of ESBL/pAmpC-producing *E. coli* isolates was calculated by dividing the number of base pair (bp) obtained for each strain over the number of bp in the reference *E. coli* genome (NC_008563.1).

	MinION					Illumina			
Sample Name	Read Length N50 (bp)	Mean Quality Read (Q)	Number of Reads	Total (bp)	Coverage	Number of Reads	Total (bp)	Mean Read Quality	Coverage
EC-7	2950	10.4	241,235	295,518,677	58.15	2,124,877	318,731,588	32.2	62.72
EC-33	1019	17.9	1,723,479	792,066,859	155.9	8,684,972	1,302,745,812	38.9	256.3
EC-40	3802	10.6	276,487	443,816,606	87.3	6,253,992	938,098,804	37.1	184.6
EC-56	7930	10.5	173,356	377,261,099	74.2	9,190,283	1,378,542,592	37.9	271.3
EC-78	1826	10.5	292,564	293,709,850	57.8	5,779,896	866,984,404	38.0	170.6
EC-91	4880	10.6	584,008	1,252,722,910	246.5	9,143,314	1,371,497,130	37.7	269.9
EC-94	3506	10.5	271,096	506,846,144	99.7	7,078,484	1,061,772,709	37.5	208.9
EC-115	1018	10.5	304,842	216,993,948	42.7	9,640,675	1,446,101,361	37.9	284.6
Mean	3366.4	11.4	483,383.4	522,367,011.6	102.8	7,237,062	1,085,559,300	36.9	213.6
SD	2295.9	2.6	515,319.1	344,470,921.0	67.8	2,523,376.8	378,506,526.8	1.8	74.5

**Table 3:** Plasmids typing according to Illumina, MinION, and hybrid assemblies.

	Illumina				MinION				Hybrid			
			MLST				MLST				MLST	
Sample Name	IncType	Circular	pST	CC	IncType	Circular	pST	CC	IncType	Circular	pST	CC
EC-7	IncA/C2	N	3	-	IncA/C2	Y	3	NA	IncA/C2	Y	3	-
EC-33	IncX1	Y	NA	NA	IncX1	Y	NA	NA	IncX1	Y	NA	NA
EC-40	IncI1	N	26	2	IncI1	N	26 *	NA	IncI1	N	New	26
EC-56	IncI2	N	NA	NA	IncI2	N	NA	NA	IncI2	Y	NA	NA
EC-78	IncHI2	N	4	-	IncHI2	N	11 *	NA	IncHI2	Y	4	-
EC-91	IncY	N	NA	NA	IncY	N	NA	NA	IncY	N	NA	NA
EC-94	Unknown	N	NA	NA	IncFII	N	Failed	Failed	IncFII	N	Failed	Failed
EC-115	IncI1	N	80	31	IncFI1	N	New *	NA	IncI1	N	80	31

* Mismatches to reference sequences.

**Table 4:** Insertion sequences (ISs) and transposons (Tns) associated with ESBL/pAmpC genes and other ARGs identified in the eight ESBL/pAmpC plasmids using Illumina, MinION, and hybrid assemblies.

		Illumina			MinION			Hybrid	
Sample Name	ESBL/pAmpC	IS/Tn	Other ARGs	ESBL/pAmpC	IS/Tn	Other ARGs	ESBL/pAmpC	IS/Tn	Other ARGs
EC-7	*bla_CMY-2_*	ISEc9	tet(A), sul2, aph(6)-Id, floR, aph(3”), qacE, sul1, aac(3)-Vla, qacL, sul3 *, aadA2b *, cmlA1 *	*bla_CMY-2_*	ISEc9	*tet(A), sul2, aph(6)-Id, qacE, floR, aadA1, aph(3”), sul1, aac(3)-Vla*	*bla_CMY-2_*	ISEc9	*tet(A), sul2, aph(6)-Id, qacE, florR, aadA1, aph(3”)-Ib, sul1, aac(3)-Vla*
EC-33	*bla_TEM-52B_*	Tn2	*none*	*bla_TEM-52B_*	Tn2	*none*	*bla_TEM-52B_*	Tn2	*none*
EC-40	*bla_SHV-12_*	IS26	*sul3, aadA1, qacL, aadA2b, cmlA, tet(A)*	*bla_SHV-12_*	IS26	*sul3, aadA1, tet(A), qacL, aadA2b, cmla1*	*bla_SHV-12_*	IS26	*sul3, aadA1, tet(A), qacL, aadA2b, cmla1*
EC-56	*bla_CTX-M-1_*	None	*none*	*bla_CTX-M-1_*	IS102	*none*	*bla_CTX-M-1_*	IS102	*none*
EC-78	*bla_CTX-M-2_*	None	*dfrA1, aph(6)-Id, qacE, sul1, tet(A), aph(3”)-Ib, aadA1, sul2, bla_TEM-1_*	*bla_CTX-M-2_*	IS26	*dfrA1, aadA1, aph(6)-Id, qacE, sul1, tet(A), aph(3”)-Ib, sul2, bla_TEM-1_*	*bla_CTX-M-2_*	IS26	*dfrA1, aadA1, aph(6)-Id, qacE, sul1, tet(A), aph(3”)-Ib, sul2, bla_TEM-1_*
EC-91	*bla_CTX-M-15_*	ISEc9	*bla_TEM-1_, sul2, aph(6)-Id, aph(3”)-Ib, qnrS1, tet(A)*	*bla_CTX-M-15_*	ISEc9	*bla_TEM-1_, sul2, aph(6)-Id, aph(3”)-Ib, qnrS1, tet(A)*	*bla_CTX-M-15_*	ISEc9	*bla_TEM-1_, sul2, aph(6)-Id, aph(3”)-Ib, qnrS1, tet(A)*
EC-94	*bla_CTX-M-1_*	None	*mph(A)*	*bla_CTX-M-1_*	Cn_5325_IS26	*mph(A)*	*bla_CTX-M-1_*	Cn_5325_IS26	*mph(A)*
EC-115	*bla_CTX-M-1_*	None	*aac(3)-IId, dfrA14*	*bla_CTX-M-1_*	IS26	*dfrA14, aac(3)-IId*	*bla_CTX-M-1_*	IS26	*dfrA14, aac(3)-IId*

* Antimicrobial Resistance Genes identified only by short read assemblies.

## Data Availability

Sequencing data were deposited in NCBI and are publicly available at PRJNA1328595.

## Supplementary Materials

The following supporting information can be downloaded at https://www.mdpi.com/article/10.3390/pathogens14101039/s1), Figure S1. (A) Tukey box plots of total length of nonESBL/pAmpC plasmids. Bar plots of (B) circular and (C) typed nonESBL/pAmpC plasmids. Tukey box plots of number of (D) ARGs, (E) IS and Tn sequences, and (F) virulence genes identified in nonESBL/pAmpC plasmids by Illumina short-reads, MinION long-reads, and hybrid assemblies.

## Abbreviations

The following abbreviations are used in this manuscript:

3GCs	Third-generation cephalosporins
AMR	Antimicrobial resistance
AmpCs	AmpC β-lactamases
ARGs	Antimicrobial resistance genes
bp	Base pairs
CC	Clonal complex
CDS	Coding DNA sequence
CI	Confidence interval
CLSI	Clinical and Laboratory Standards Institute
CTX-EMB	Cefotaxime Eosin Methylene Blue
*E. coli*	*Escherichia coli*
EMB	Eosin Methylene Blue
ESBLs	Extended-spectrum β-lactamases
HGT	Horizontal gene transfer
IS	Insertion sequence
kb	Kilobase pairs
MGEs	Mobile genetic elements
N50	Assembly contiguity statistic
NA	Not assigned/applicable
ONT	Oxford Nanopore Technologies
PCR	Polymerase chain reaction
pMLST	Plasmid multilocus sequence typing
pST	Plasmid sequence type
Q	Quality score (Phred)
SD	Standard deviation
ST	Sequence type
Tn	Transposon
VF	Virulence factor
WGS	Whole-genome sequencing
