# Supplementary material for: Deciphering Escherichia coli ESBL/pAmpC Plasmids Through High-Throughput Third-Generation Sequencing and Hybrid Assembly
Source: Pathogens. 2025 Oct 13;14(10):1039. doi: 10.3390/pathogens14101039 (PMC12567533; doi:10.3390/pathogens14101039)

Figure S1. (**a**) tukey box plots of total length of nonESBL/pAampC plasmids. Bar plots of (**b**) circular and (**c**) typed nonESBL/pAampC plasmids. Tukey box plots of number of (**d**) ARGs, e) IS and Tn sequences, and f) virulence genes identified in nonESBL/pAampC plasmids by Illumina short-reads (blue), MinION long-reads (red), and hybrid assemblies (green). $p < 0.05$ is shown as

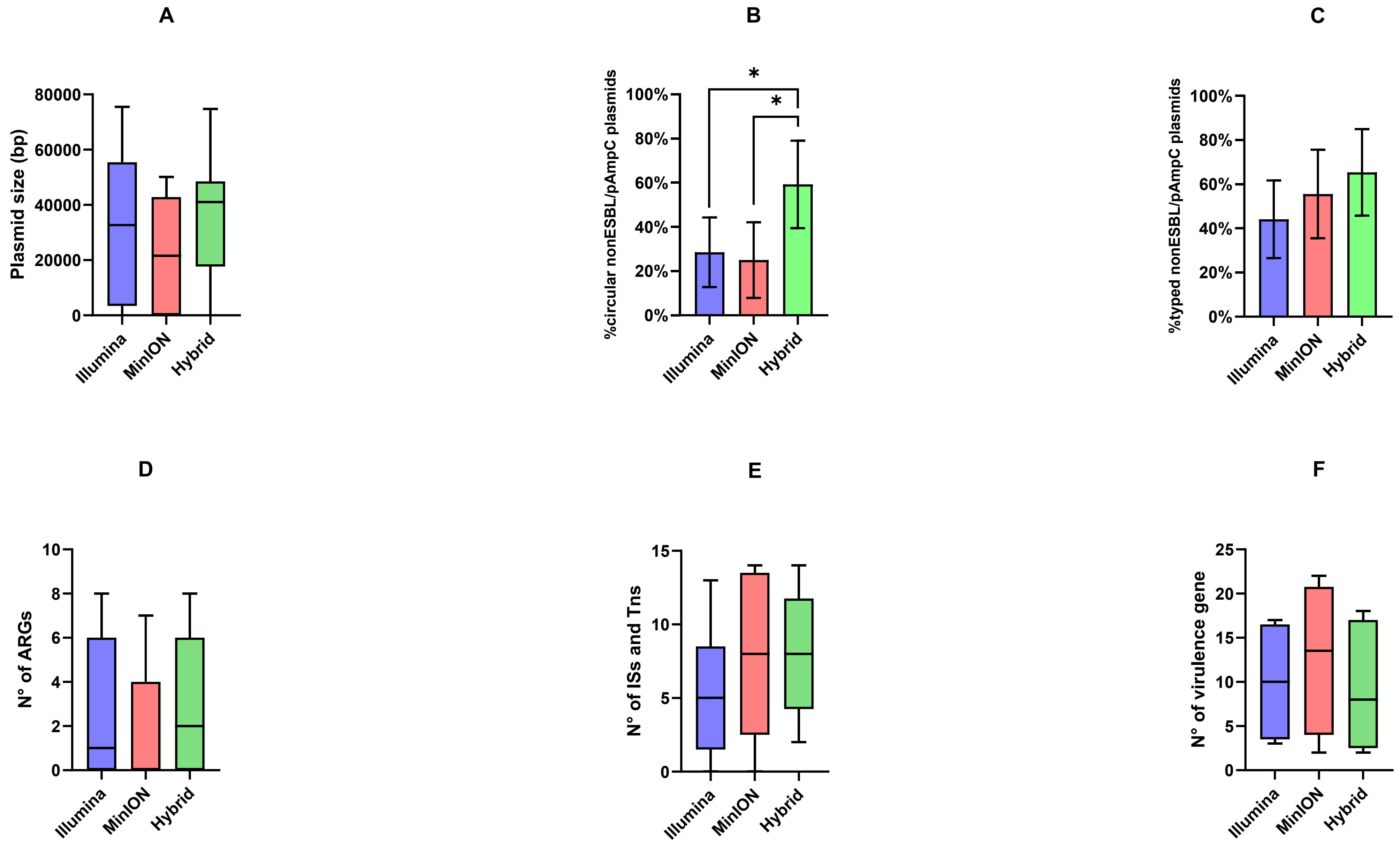

Supplement: Supplementary file 1 [file pathogens-14-01039-s001.zip › pathogens-3904010-supplementary.pdf]
